# Comparing ERAS-outpatient versus standard-inpatient hip and knee replacements: a mixed methods study exploring the experience of patients who underwent both

**DOI:** 10.1186/s12891-021-04847-9

**Published:** 2021-11-23

**Authors:** Alexandre Hardy, Jonathan Gervais-Hupé, François Desmeules, Anne Hudon, Kadija Perreault, Pascal-André Vendittoli

**Affiliations:** 1 Department of Biomedical Sciences, Faculty of Graduate and Postdoctoral Studies, Université de Montréal, Montreal, Quebec Canada; 2 Hôpital Maisonneuve-Rosemont, Surgery Department, Université de Montréal, Montreal, Quebec Canada; 3 Centre de recherche de l’Hôpital Maisonneuve-Rosemont, Quebec Montreal, Canada; 4 School of Rehabilitation, Faculty of Medicine, Université de Montréal, Montreal, Quebec Canada; 5 Centre de recherche interdisciplinaire en réadaptation du Montréal métropolitain (CRIR), Montreal, Quebec Canada; 6 Centre de recherche en éthique (CRÉ), Université de Montréal, Montreal, Quebec Canada; 7 Department of Rehabilitation, Faculty of Medicine, Université Laval, Quebec, Canada; 8 Center for Interdisciplinary Research in Rehabilitation and Social Integration (Cirris), Quebec, Canada; 9 Personalized Arthroplasty Society, Atlanta, Georgia USA; 10 Duval Orthopaedic Clinic, Laval, Quebec Canada

**Keywords:** Arthroplasty, replacement, knee, Arthroplasty, replacement, hip, Enhanced recovery after surgery, Fast-track, Outpatient, Patient experience, Patient outcome assessment, Mixed methods research

## Abstract

**Background:**

Optimizing patients’ total hip and knee arthroplasty (THA/TKA) experience is as crucial for providing high quality care as improving safety and clinical effectiveness. Yet, little evidence is available on patient experience in standard-inpatient and enhanced recovery after surgery (ERAS)-outpatient programs. Therefore, this study aimed to gain a more in-depth understanding of the patient experience of ERAS-outpatient programs in comparison to standard-inpatient programs.

**Methods:**

We conducted a convergent mixed methods study of 48 consecutive patients who experienced both standard-inpatient and ERAS-outpatient THA/TKA contralaterally. A reflective thematic analysis was conducted based on data collected via a questionnaire. Bivariate correlations between the patient experience and patients’ characteristics, clinical outcomes and care components satisfaction were performed. Then, the quantitative and qualitative data were integrated together.

**Results:**

The theme *Support makes the difference for better and for worse* was identified by patients as crucial to their experience in both joint replacement programs. On the other hand, patients identified 3 themes distinguishing their ERAS-outpatient from their standard-inpatient experience: 1) *Minimizing inconvenience*, *2) Home sweet home* and 3) *Returning to normal function and activities.* Potential optimization expressed by patients were to receive more preoperative information, additional postoperative rehabilitation sessions, and ensuring better coherence of care between hospital and home care teams. Weak to moderate positive and statistically significant correlations were found between patients’ THA/TKA experience and satisfaction with pain management, hospital stay, postoperative recovery, home care, and overall results (r_s_ = + [0.36–0.66], *p*-value < 0.01).

**Conclusion:**

Whatever the perioperative program, the key to improving patients’ THA/TKA experience lies in improving support throughout the care episode. However, compared to standard-inpatient care, the ERAS-outpatient program improves patients’ experience by providing dedicated support in postoperative care, reducing postoperative inconvenience, optimizing pain management, returning home sooner, and recovering and regaining function sooner. Patients’ THA/TKA experience could further be enhanced by optimizing the information provided to the patient, the rehabilitation program and the coherence between care teams.

**Supplementary Information:**

The online version contains supplementary material available at 10.1186/s12891-021-04847-9.

## Introduction

Fast-track protocols represent a potential solution to the challenges posed by the expected increase in demand for total hip and knee arthroplasty (THA/TKA) over the coming decades and wait times exacerbated by the COVID pandemic [1–3]. The shift from inpatient to outpatient programs for THA/TKA is also attractive due to increased capacity and bed availability, as well as reduced length of stay and health care costs [4–6]. However, the main objective for transitioning to an ambulatory practice should be to improve the recovery process to a level where it is safe for the patients to return home on the day of surgery [7]. To achieve this objective, outpatient programs should be based on enhanced recovery after surgery (ERAS) principles that focus on optimizing all aspects of perioperative care to ensure effective, secure and rapid recovery [8, 9]. To offer high-quality care, ERAS-outpatient THA/TKA aim to optimize the three pillars of quality of care as described by the National Health Service: patient safety, effectiveness of care and patient experience [10, 11].

A recent study by Hardy & al [12]. compared a THA/TKA ERAS-outpatient to standard-inpatient care on all three pillars and found that the ERAS-outpatient reduced complications and opioid consumption, enabled faster functional recovery and improved patients’ satisfaction and experience on a visual analog scale. Other studies corroborated these findings but yet, very little evidence is available on patient experience [13–17]. Patient experience is a complex concept, representing “the sum of all interactions, shaped by an organization’s culture, that influence patient perceptions, across the continuum of care” and that understanding the patient experience or factors enhancing it, goes beyond quantitative assessment alone [18, 19]. Therefore, this study’s overarching objective was to gain a more in-depth understanding of the patient experience for both STD-inpatient and ERAS-outpatient THA/TKA by combining the strengths of quantitative and qualitative approaches [20]. Specifically, in subjects who experienced both an ERAS-outpatient and a STD-inpatient program, we wanted to compare their experience and identify elements that could be optimized. We also sought to determine whether and how patient characteristics, clinical outcomes and satisfaction of care components are associated with patients’ experience.

## Methods

### Study design, patient selection and characteristics

This study used a convergent mixed methods single subject design (Quan + Qual) to gain a more in-depth understanding of patient experience for both STD-inpatient and ERAS-outpatient THA/TKA. In October 2020, in conjunction with Hardy & al. study [12], we recruited the first 50 adult patients who sequentially underwent both programs for the same primary surgery on contralateral articulations in a Canadian tertiary hospital and who were able to communicate in French or English. Out of all eligible participants, 48 gave an informed written consent and enrolled in the study; two patients declined because they were not interested in participating. The criteria patients had to fit to undergo the ERAS-outpatient program were described by Vendittoli et al. [13] The study was conducted in accordance with the Declaration of Helsinki and this study was approved by our Institutional Review Board (*Comité d’éthique de la recherche du CIUSSS de l’Est-de-l’Île-de-Montréal* #2021–2420). Full details of participants’ characteristics were presented in a previous paper [12] and are summarized in Table 1.

**Table 1:** Patients’ Characteristics

	ERAS-outpatient	STD-Inpatient	***P***-value
**Number of participants**	48		
**Gender**			
(Men: Women)	27 (56.2%): 21 (43.8%)		
**Average Age at Surgery**	60.0 (45.0–76.0,7.8)	52.9 (33.0–70.0, 8.3)	< 0.001^a^
**Average Body Mass Index**	28.1 (20.7–37.6, 4.1)	28.3 (19.3–35.0, 4.0)	0.638^a^
**ASA Physical Status**			0.007^b^
1	17 (35.4%)	25 (52.1%)	
2	30 (62.5%)	23 (47.9%)	
3	1 (2.1%)	0	
**Intervention**			
THA	36 (75.0%)		
TKA	12 (25.0%)		

^a^Paired T-test

^b^Marginal homogeneity test

### STD-inpatient and ERAS-outpatient programs description

In both programs, patients met with several health professionals weeks before the day of surgery and an additional informative group session was offered in the ERAS-outpatient program. Recommendations before surgery were the same for both programs except that fasting after midnight was required for STD-inpatient, whereas they could drink clear liquids up to 2 h before the ERAS-outpatient operation. Perioperative care provided in the STD-inpatient program varied according to the patient’s characteristics and surgeon’s preferences, whereas it was standardized for ERAS-outpatient (every patient received the same interventions). Surgical approach and implants were identical for both programs. The first physiotherapy session aimed to be performed on postoperative day 1 for STD-inpatient and in the first 4–6 h after surgery for ERAS-outpatient. STD-inpatient patients were expected to be hospitalized 1–3 days for a THA and 3–5 days for a TKA whereas ERAS-outpatient cases were expected to be discharged the same day of surgery (THA) or in < 24 h (TKA). Regardless of the program, all patients returned home after discharge, followed the same rehabilitation program, and received similar home care services. STD-inpatient surgeries took place between 2000 and 2018 and were all performed before ERAS-outpatient surgeries, performed between 2017 and 2020. Detailed description of both ERAS-outpatient and STD-inpatient programs were previously presented by Vendittoli et al. [13] and Hardy et al. [12]

### Data collection

Patient characteristics and postoperative data: pain on a numeric rating scale (NRS) [0–10], opioid consumption in milligram morphine equivalent, numbers of complications measured with the Clavien-Dindo classification, complications’ morbidity rated with the Comprehensive Complication Index, numbers of unplanned episodes of care, length of stay in hours, and time in days to reach functional recovery landmarks at the hospital were collected retrospectively from medical records. Through the *Patient Experience* questionnaire [Supplementary Material: Appendix A], we collected prospectively, at the last follow-up (9 months to several years after surgeries): the time to reach functional recovery landmarks at home, patient overall care experience, and patient satisfaction of care components (preparation before surgery, hospital stay, home care, pain management, postoperative recovery, wound care, overall result) on visual analog scale (VAS) [0–100]. When patient overall care experience and patient satisfaction of care components on VAS differed between programs, patients were asked open-ended questions to provide a deeper understanding of their experience. In addition, participants were invited to explain their answers when questioned about which program they would recommend. Moreover, they were invited to suggest any improvement they thought that could be made to the programs to enhance their experience. To prevent any potential wording bias related to the names of the programs “Enhanced Recovery after Surgery” versus “Standard”, all questions referred to “Right” and “Left” sides instead. Once the survey completed, answers were categorized to the appropriate program (ERAS-outpatient or STD-inpatient) before proceeding to analyses. All the data collected were de-identified and stored in a secured REDCap database [Research Electronic Data Capture, Vanderbilt University, TN, USA].

### Data processing and analysis

#### Qualitative data

All text data gathered through open-ended questions in the *Patient Experience* questionnaire were qualitatively analyzed in QDA Miner [QDA Miner, Version 5.0, Provalis Research, Montreal, QC, CAN] using a reflexive thematic analysis inspired by Braun & Clark [21]. First, two authors (A. Hardy and J.G.H.) read the entire data set many times to become familiar with it. They recorded their thoughts in a research audit trail. Second, based on an inductive and semantic approach, both investigators generated initial codes independently for five participants. Then, they compared their initial codes, made modifications if needed, and elaborated a common codebook based on their consensus. Third, they repeated this process for five more participants to assure trustworthiness regarding the analytical approach. Fourth, A. Hardy coded the data of remaining participants, and afterwards grouped codes into broader categories and developed overarching themes. Fifth, he created a thematic map to visualize the relationships between themes and to sort main themes from secondary ones. Sixth, the representation of results was debriefed and discussed with all investigators which led A. Hardy to reread the entire data set and refined themes to form a coherent whole that truly captured the essence of the participants’ experiences. When refinements stopped producing significant improvements, final themes were approved by the whole research team.

#### Quantitative data

Relations between patient overall care experience on the VAS [0–100] and continuous data on patient characteristics, clinical outcomes and patient satisfaction of care components were analyzed with Pearson or Spearman bivariate correlations. Point-biserial correlations were realized when patient characteristics and clinical outcomes data were dichotomous. A two-sided alpha level of significance of 0.05 was used and all tests were performed with *SPSS - 25* [IBM SPSS Statistics for Macintosh, Version 25.0. IBM Corp Armonk, NY, USA].

#### Quantitative and qualitative data integration

The qualitative and quantitative data were first analyzed separately. Then, they were integrated together in the discussion and compared to the literature to provide a more in-depth understanding of patient experience for both STD-inpatient and ERAS-outpatient THA/TKA [22].

## Results

### Qualitative results

The qualitative analysis allowed to identify themes that provided a better understanding of the patient experience during THA/TKA. One theme was commonly present among both programs: *Support makes the difference for better and for worse.* Three other themes captured improvements in the patient experience due to the ERAS-outpatient program compared to STD-inpatient one: 1) *Minimizing inconvenience*, *2) Home sweet home* and 3) *Returning to normal function and activities*. Finally, another theme captured improvements that could be made to the patient experience*: Room for improvement***.** Quotes from Francophone participants were translated into English by a native English speaker.

#### Support makes the difference for better and for worse

No matter the phase of care and the program, participants spontaneously evoked their appreciation for benefiting from a well-framed structure from beginning to end. They felt better supported and confident when healthcare providers identified themselves and answered their questions. They appreciated healthcare providers who are good listeners and valued receiving clear information, so they were not left in the unknown.

*“For me, everything was perfect, the information was clear, the care from start to finish, everything was well managed, I never felt in the unknown.”* (Patient 40).

On the other hand, they were dissatisfied when the first contact with the healthcare team was judged as non-professional or when their surgeon did not visit them after the intervention. Participants also discussed how the support provided on the ward after surgery affected their experience. Some disliked their STD-inpatient experience because they thought that postoperative care was disorganized and of poor quality. They observed that the staff seemed overwhelmed and felt that they did not care about them as several patients overheard the staff arguing about who will assume responsibility for their care. Others shared how they felt let down because they experienced complications and healthcare providers took a long time to acknowledge and address them. In contrast, other participants appreciated the competency and professionalism of healthcare workers dedicated to the outpatient ward and some even expressed they felt pampered. Participants also discussed the support they received at home after surgeries. Most of them enjoyed the home care services provided by nurses and physiotherapists in both programs. They said they appreciated the professionalism of home care professionals as they took all the time needed to provide care, answer questions and to refer to other healthcare professionals when required. Some participants even said that having a dedicated member of the orthopaedic team made available to answer their questions and tamper their fears and anxieties was a huge plus. On the other hand, a few participants had mixed feelings about the quality of care they received. These participants considered that the healthcare workers who came to assist them were incompetent and unprofessional. They stated that home care providers changed personnel often, were not familiar with their file, did not follow the instructions given by the surgical team, did not detect complications, were unable to answer their inquiries and sometimes did not show up to provide the prescribed care. Overall, patients expressed the importance of support from the healthcare team throughout the entire care process in both programs. However, when participants reported differences between programs, they usually felt better supported in the ERAS-outpatient program because they benefited from workers dedicated to this program in the ward.

*“Left hip, day surgery protocol. AWESOME! Qualified and dedicated staff for day surgeries. Always someone close by to make sure everything goes well. I had questions about my medication, the orthopaedic nurse referred me to a pharmacist right away and I quickly got the right advice. I felt like I was being treated like a princess because the people were so attentive.”* (Patient 34).

#### Minimizing inconvenience

Patients noted differences between programs regarding prevention and management of adverse events. For pain management, participants preferred the ERAS-outpatient program because their pain was better relieved with less medication and being home sooner allowed them to take analgesics as prescribed without having to wait for the hospital staff as during STD-inpatient. Patients also liked the opioid-sparing analgesic modalities of the ERAS-outpatient because it prevented side effects associated with opioids, including vomiting or constipation, and alleviated their fear of developing an addiction. Moreover, patients noted that staples used to close the surgical wound were replaced by sutures and tissue adhesive in the ERAS-outpatient program which eliminated their bothersome side effects like wound discharge and itching in addition to the inconvenience and pain of having staples removed. Many participants also preferred the use of tissue adhesive (ERAS-outpatient) because it enabled them to shower sooner and they felt that their wounds healed better and more quickly, resulting in smaller and more aesthetical scar.

*“With the glue, it’s fantastic, no discharge, you can shower faster, the scar heals faster and looks better”* (Patient 40).

Overall, patients reported the type of anesthesia as being a major difference between the two programs. Some stated that, after ERAS-outpatient, they did not suffer from prolonged motor blockade nor from urinary retention unlike during STD-inpatient. Others reported that the epidural-sedation anesthesia used in ERAS-outpatient was as efficient as the spinal anesthesia used in STD-inpatient, but better tolerated with fewer side effects. They added that feeling less sedated enabled waking up and recovering more quickly after the surgery.

*“Easier to wake up from surgery* (ERAS-outpatient) *and go home the next day, less sick. I was much better in general when I came out of the operating room than at the first operation* (STD-inpatient)*.”* (Patient 10).

Globally, participants expressed that postoperative inconveniences were better prevented or managed with ERAS-outpatient.

#### Home sweet home

For some patients, the expectation to go home only hours after surgery was at first a source of anxiety. Nevertheless, they were reassured because a caregiver would be with them. Because they felt the ward can be a harsh setting in which to recover, most patients appreciated the reduced hospital length of stay with ERAS-outpatient.

*“The less time spent in hospital the better! Long live day surgery!”* (Patient 45).

They evoked the difficulty of getting a good night of sleep because of the noise, their lack of interest in the hospital food and the anxiety-inducing atmosphere of the environment. Even when they reported excellent care at the hospital for their first surgery, patients appreciated leaving the hospital as early as possible and found convalescence was easier and better for the mind at home.

*“Despite the good care of the nursing staff, recovery is easier at home, in my environment, much easier and better for the spirit.” (Patient 25).*

#### Returning to Normal function and activities

Most participants reported major differences between programs in the time and effort required to return to normal function. With ERAS-outpatient, they expressed their delight in performing activities of daily living, such as walking and climbing stairs on the day of surgery, which was not the case after STD-inpatient. Some even shared their astonishment in being able to go shopping and going back to work only a couple of days later.

*“For the left leg with the day surgery, the recovery was really superfast. 3 days later I was at the grocery shop and the next day I was going to the office! Much faster than the first operation which still went very well in terms of recovery.”* (Patient 45).

Participants noted that the overall recovery process was generally quicker and easier following the ERAS-outpatient THA/TKA because of what they perceived as enhanced supervision and better adapted exercises. They noted that fewer physiotherapy sessions were needed to recover compared to STD-inpatient.

*“For the right side* (STD-inpatient) *much more time in physio 1–2 months, whereas the left side* (ERAS-outpatient) *3 visits in physio and finished thereafter.”* (Patient 3).

However, in both programs, some patients experienced obstacles such as pain and deficits in strength and flexibility that hampered their functional recovery. For very few patients, these difficulties persisted and negatively tainted their experience, whereas most patients were happy with the overall results of both THA/TKA.

#### Room for improvement

Improvements to THA/TKA programs were highlighted by some patients. For the preparation phase, patients suggested to present patients’ testimonies of previous experiences and to further explain the patient’s role in the process to optimize recovery. They further recommended that surgeons come visit their patients in the recovery room. Moreover, patients suggested that having more physiotherapy sessions, as needed, would be beneficial.

“*Having more physiotherapy at home if needed: I believe this is the key after a successful surgery*” (Patient 8).

Also, participants recommended that communication between the hospital team and home care teams be improved to convey more coherent instructions.

*“Improved communication between the hospital physio and the CLSC* 1*physio. The hospital physio explained to me that with the new protocol (ERAS-outpatient) I could start walking, climbing stairs and even riding a stationary bike faster whereas the CLSC asked me to wait.”* (Patient 1).

Finally, they advocated to maintain having a dedicated resource person from the hospital team easily reachable by phone to answer inquiries.

### Quantitative results

The bivariate correlation between patients’ overall care experience and patient characteristics and clinical outcomes showed only poor association r_s_ = ± [.000–0.299] that was not statistically significant (*p* > 0.05; Table 2). The positive associations between patients’ overall care experience and satisfaction of care components in the ERAS-outpatient were found to be fair r_s_ = + [0.400–0.599] to moderate r_s_ + [0.600–0.799] and were all considered statistically significant (*p* < 0.01; Table 3). The spearman correlation coefficients between these same variables in the STD-inpatient showed generally weaker positive associations, ranging from poor and non-statistically significant (p ≥ 0.108) for preparation and wound care to fair and statistically significant for pain management, postoperative recovery, hospital stay, home care and overall results (*p* < 0.014).

**Table 2:** Relations Between Patient Overall Care Experience and Patients’ Characteristics and Clinical Outcomes

Patient characteristics	ERAS-Outpatient		STD-Inpatient	
	Spearman correlation coefficient	***P***-value	Spearman correlation coefficient	***P***-value
Gender	.044	0.771	.137	0.359
Age at surgery	.148	0.321	.158	0.288
Body Mass Index	.152	0.309	.136	0.368
ASA Physical Status	.163	0.273	.089	0.553
Intervention (THA/TKA)	.091	0.545	−.188	0.205
**Postoperative variables**				
Pain in the recovery room (NRS 0–10)	.002	0.99	−.071	0.651
Pain in the ward, surgery day (NRS 0–10)	.235	0.112	−.043	0.779
Mean Opioid Consumption in Morphine Milligram Equivalents in the First 8 Hours After Surgery	−.163	0.275	−.087	0.56
Complications: All Grades	.060	0.689	.020	0.892
Complications: Grade 1	.053	0.721	.085	0.572
Complications: Grade 2	−.037	0.806	−.273	0.063
Comprehensive Complication Index	−.023	0.876	−.163	0.273
Emergency Care Unit visits without intervention	−.239	0.106	−.039	0.794
Emergency Care Unit visits with interventions	−.015	0.922	.022	0.882
Clinic consultations without intervention	−.057	0.706	.034	0.822
Clinic consultations with interventions			−.257	0.082
First rise/standing (in days)	.232	0.117	.192	0.218
Walking (in days)	.058	0.71	.144	0.382
Going up and down the stairs (in days)	.205	0.181	−.003	0.985
Length of stay (in hours)	.095	0.527	−.162	0.281
First shower (in days)	−.103	0.497	−.245	0.104
Walking without technical aid (in days)	−.005	0.972	−.106	0.484
Going up and down the stairs without technical aid (in days)	−.014	0.923	−.031	0.836
ADLs: dressing, toileting, walking indoor alone, etc. (in days)	−.19	0.201	−.174	0.248
iADLs: cooking, cleaning, shopping, etc. (in days)	−.007	0.965	−.109	0.472
Mild physical activities: cycling, swimming, walking, etc. (in weeks)	−.085	0.584	.088	0.567
Intense physical activities: running, playing tennis, skiing, etc. (in weeks)	.28	0.166	.106	0.59
Return to light-duty work (in weeks)	.285	0.121	.097	0.562
Return to regular work without limitation (in weeks)	.193	0.344	.069	0.707

**Table 3:** Relations Between Patient Overall Care Experience and Satisfaction of Care Components (VAS 0–100)

Variables	Patient Overall Care Experience and Satisfaction of care components (VAS 0–100) [12]			ERAS-outpatient		STD-inpatient	
	ERAS-outpatient Mean (Min-Max, SD)	STD-inpatient Mean (Min-Max, SD)	***P***-value	Spearman correlation coefficient	***P***-value	Spearman correlation coefficient	***P***-value
Patient overall care experience	97 (75–100, 30)	88 (24–100, 16)	< 0.001				
Satisfaction with preparation	94 (60–100, 10)	93 (53–100,12)	0.356	.453	0.001	0.238	0.108
Satisfaction with hospital stay	96 (50–100, 9)	85 (0–100, 23)	< 0.001	.614	< 0.001	.450	0.001
Satisfaction with home care	89 (11–100, 20)	92 (39–100, 13)	0.678	.661	< 0.001	.411	0.004
Satisfaction with pain management	93 (18–100, 14)	87 (0–100, 21)	0.002	.582	< 0.001	.357	0.014
Satisfaction with functional recovery	95 (70–100, 7)	84 (1–100, 20)	< 0.001	.486	0.001	.374	0.01
Satisfaction with wound management	95 (18–100, 13)	85 (0–100, 18)	< 0.001	.614	< 0.001	0.124	0.405
Satisfaction with overall result	96 (70–100, 8)	92 (0–100, 17)	0.187	.578	< 0.001	.420	0.003

## Discussion

The qualitative analysis demonstrated that the quality of support received by the patients throughout the episode of care is important to their THA/TKA experience, and that overall, patients had a better experience with the ERAS-outpatient program because they felt better supported by staff dedicated to ambulatory surgery in the ward, they experienced less postoperative inconvenience, went home sooner, and recovered more quickly. Furthermore, patients suggested that their THA/TKA experience could possibly be enhanced by improving the information given in the preparation phase, providing more postoperative physiotherapy sessions at home if needed and ensuring better coherence of care between hospital and home care teams. The bivariate analyzes revealed a weak to moderate positive correlations between patient experience and satisfaction with pain management, postoperative recovery, hospital stay, home care and overall results. Taken separately, these findings enhance our comprehension of the patient THA/TKA experience. Nevertheless, this study’s overarching objective was to gain a more in-depth understanding of the patient experience for both STD-inpatient and ERAS-outpatient THA/TKA by combining the strengths of quantitative and qualitative approaches [20]. Consequently, qualitative and quantitative results were integrated together and compared to the literature in the following paragraphs.

### Patients characteristics

Bivariate analyses did not find relationship between patients’ characteristics and their overall care experience nor was it raised by the qualitative analysis. Taken together, these findings suggest that patients’ characteristics are not associated with their overall care experience and that the enhanced patient experience in the ERAS-outpatient programs was unlikely to be associated with the difference in patients’ age and comorbidities between surgeries.

### Clinical outcomes

In this cohort of patients, it was shown that compared to the STD-inpatient, the ERAS-outpatient program resulted in fewer complications, similar postoperative pain with less opioid consumption and sooner functional recovery [12]. These findings were corroborated by the present qualitative analysis as patients clearly discussed how clinical outcomes, such as adverse events, pain, and functional recovery, affected their experience. However similarly to other studies, no significant statistical correlation between clinical outcomes and the patient experience was found [23–25]. These divergent quantitative and qualitative findings underscored the importance of going beyond quantitative assessment alone to truly understand what influences and how it affects the patients’ care experience.

### Postoperative recovery

Sooner and better recovery after surgery is the main goal of ERAS interventions [26]. The resulting optimized recovery was a major reason why patients reported a better THA/TKA experience with ERAS-outpatient. The ERAS-outpatient epidural-sedation combination had the advantage over spinal anesthesia of eliminating the distress associated with being awake during the operation and contributed to make patients feel better and recover more quickly after surgery [27, 28]. The earlier ambulation after surgery contributed to reduce complications and to improve pain, muscles strength, range of motion, gait and overall function in the post-acute phase which may explain why patients enjoyed their ERAS-outpatient recovery, even though the exercise program was similar [16, 29–31]. The earlier ambulation also helped reduce the number of rehabilitation sessions needed to reach functional autonomy which was greatly appreciated by the patients [12, 29]. Thus, by improving the recovery process, the ERAS-outpatient program optimized the overall patients’ experience. Inversely, Johansson Stark et al. [32] observed that a positive patient experience increased the likelihood of better postoperative recovery. Consequently, as recommended by Doyle et al. [11], all three pillars of quality of care (patient safety, clinical effectiveness and patient experience) should be viewed as a group and interventions should aim to improve all three to provide high-quality care in THA/TKA programs.

### Pain management

Pain management is crucial after THA/TKA, as unrelieved pain can negatively affect recovery and patient experience [33–37]. With outpatient programs, patients become quickly responsible for their pain management and it was shown to often result in unrelieved pain [34, 38]. When self-medicating, patients are often reluctant to take the prescribed analgesics because they are not sure when to take them or because they are afraid of developing an addiction [34, 35, 38]. This highlights the importance of educating patients so that they can be confident in the postoperative process and demonstrate the necessary coping skills [39]. Sharing advice from former patients could further empower patients in the process of care, thereby increasing their confidence in managing their pain appropriately [40–42]. The pre-discharge education provided in the ERAS-outpatient program was key to make participants appreciated being able to self-manage their consumption of opioid-sparing analgesics, as this allowed them to relieve their pain quickly, without having to wait for nursing staff and with less fear of developing a dependency. This method of self-medication, when applied appropriately, has been shown to be strongly associated with better overall pain relief, less need for additional analgesia and, therefore, a better care experience [43].

### Hospital stay and home care

The quality of hospital and home care is important to the overall patients’ experience and their satisfaction with these components of care is positively associated to it. The patients appreciated care providers who were competent, engaged and interested in helping them recover, and recognized the added value of being cared by nurses and physiotherapists specialized in the ERAS-outpatient program [34]. However, a care provider deemed unprofessional or not cohesive with the medical team instructions increase patients’ uncertainty [34]. Fortunately, patients benefited from having a nurse and a physiotherapist, from the hospital team, available by phone to answer doubts, ease concerns, and provide information which helped to improve the overall provider-patient relationship [44, 45]. Additionally after ERAS-outpatient, a caregiver was present at home in the early postoperative days, to help alleviate the uncertainties of home care and temper the additional concerns of returning home quickly [35, 46]. The present findings reinforced those of McMurray et al. [45] stating that relationships between patients and care providers are a key component of the patient experience and that aspects such as caring, empathy, respect and perceived provider expertise all influenced the overall experience [45]. On the other hand, the poor home care experience by some patients underscores the importance of improving and standardizing the quality of home care and educating home care providers on the rehabilitation protocol in order to provide a consistent message that will improve patient experience and also potentially postoperative outcomes [47].

### Overall results

Satisfaction with THA/TKA overall results is positively correlated with patient overall care experience. However, patients did not express a difference between their ERAS-outpatient and STD-inpatient surgery overall results, which can be explained by the similar PROMs at the last follow-up [12]. Indeed, Black et al. [48] observed a weak positive correlation between patient-reported experience measures (PREMs) and patient-reported outcome measures (PROMs) at 6 months after a THA/TKA. Thus, while it is unlikely that satisfaction with the THA/TKA overall results contributed much to the better patient experience with the ERAS-outpatient program found in this cohort, they are still significantly related.

### Study limitations

The present results must be interpreted taking into consideration its limitations. First, while adequate, our sample size was relatively small for bivariate analyses, and they did not provide information regarding causality or direction. Nevertheless, this limitation was mostly overcome by the mixed methods design which enable to obtain a complete understanding of the patient experience and its associated factors by integrating qualitative data to the quantitative data. Second, unlike the case with interviews, our survey-based instrument did not allow us to ask sub-questions that may have allowed to further deepen the participants’ points of view. However, the large sample size by qualitative research standards was more than sufficient to gather a great variety of perspectives and experiences. Third, all surgeries were performed at the same Canadian tertiary centre and patients eligible for ERAS-outpatient had to meet specific inclusion criteria, resulting in a relatively young and healthy cohort which may limit the generalizability of the present findings to other settings or populations. Fourth, all STD-inpatient surgeries were performed before the ERAS-outpatient ones for which patients were older and more comorbid. Nevertheless, the comparison of patient satisfaction between the first and the second procedure in bilateral asynchronous TKA showed no difference and patients being older would had negatively impacted the ERAS-outpatient outcomes [49]. Fifth, participants had to recall their surgeries to answer the Patient Experience questionnaire. Yet, many strategies were put in place to minimize the impact of patient recall: participants were their own comparison, they were blind to the study hypothesis, they could take as much time as they needed to answer, data collection was standardized and done simultaneously for both programs [50].

## Conclusion

This study demonstrated that support throughout the care episode is key to the patient experience and that by reducing postoperative inconvenience, offering enhanced pain management and dedicated support in the ward, enabling to go home sooner and to recover and regain function sooner, ERAS-outpatient arthroplasty programs resulted in an optimized patient experience compared to STD-inpatient practice. Furthermore, patients’ experience of THA/TKA could benefit from optimizing the information provided, the rehabilitation program and the coherence between care teams.

## Supplementary Information

**Additional file 1.**

## Data Availability

The datasets used and/or analysed during the current study are available from the corresponding author on reasonable request.

## Abbreviations

ERAS: Enhanced Recovery after Surgery
PREMs: Patient reported experience measures
PROMs: Patient Reported Outcome Measurement Study
NRS: Numeric rating scale
THA: Total hip arthroplasty
THA/TKA: Total hip and knee arthroplasty
TKA: Total knee arthroplasty
STD: Standard

## Footnotes

1 Centres locaux de services communautaires (CLSC) are the home care providers.
