# Supplementary material for: Comparing ERAS-outpatient versus standard-inpatient hip and knee replacements: a mixed methods study exploring the experience of patients who underwent both
Source: BMC Musculoskelet Disord. 2021 Nov 23;22:978. doi: 10.1186/s12891-021-04847-9 (PMC8611950; doi:10.1186/s12891-021-04847-9)
Supplement: Supplementary file 1 — Additional file 1. [file 12891_2021_4847_MOESM1_ESM.docx]

Appendix A: Patient’s Experience

THA  TKA

Patient ID : ______________ Date : _____________________

The following questions refer to your right and left hip/knee replacements. Please answer to ALL the questions the most precisely possible.

Did you experienced complications related to your surgery? Check all that apply.

| Complication(s) | Left | Right |
| --- | --- | --- |
| None |  |  |
| Pain not relieved by the prescribed medication |  |  |
| Nausea |  |  |
| Vomitting |  |  |
| Dizziness |  |  |
| Constipation |  |  |
| Hypotension limiting mobilization |  |  |
| Infection |  |  |
| Erythema at the surgical wound |  |  |
| Veinous thrombosis (clot in a vein) |  |  |
| Fever |  |  |
| Legs edema (important swelling) |  |  |
| Headache |  |  |
| Ecchymosis black/blue/yellow discolouration of the skin) on the legs |  |  |
| Fall |  |  |
| Phlyctena (big blister) |  |  |
| Pruritus (itching) |  |  |
| Important wound discharge requiring a dressing change |  |  |
| Limping |  |  |
| Foot paralysis |  |  |
| Diaphoresis (intense sweating) |  |  |
| Urinary retention (inability to urinate) |  |  |
| Fracture |  |  |
| Abnormal bleeding |  |  |
| Heart attack |  |  |
| Stroke |  |  |
| Gastritis (inflammation of the stomach) |  |  |
| Pulmonary embolia |  |  |
| Other |  |  |

If you checked “Other” for your surgery on the LEFT side, please detail what you mean by "Other".

________________________________________________________________________________________________________________________________________________________________________________________________

If you checked “Other” for your surgery on the RIGHT side, please detail what you mean by "Other".

________________________________________________________________________________________________________________________________________________________________________________________________

**During the first two weeks after your surgery, what medication did you use to alleviate REGULARLY the pain related to your surgery? Select all that apply.**

| **Medication** | **Left** | **Right** |
| --- | --- | --- |
| None |  |  |
| Acetaminophene (Tylenol) |  |  |
| Nonsteroidal anti-inflammatory drugs (Advil, Celebrex, Naproxen) |  |  |
| Tramadol (Ultram) |  |  |
| Tramadol + Acetaminophene (Tramacet) |  |  |
| Oxycodone (Supeudol) |  |  |
| Hydromorphone (Dilaudid |  |  |
| Morphine |  |  |
| Fentanyl |  |  |
| Other |  |  |

Please detail what you mean by "Other(s)" medication(s) used to alleviate your pain during the first two weeks following your surgery on the LEFT side.

________________________________________________________________________________________________________________________________________________________________________________________________

Please detail what you mean by "Other(s)" medication(s) used to alleviate your pain during the first two weeks following your surgery on the RIGHT side.

________________________________________________________________________________________________________________________________________________________________________________________________

**For the following questions, please draw a vertical line (top to bottom) on the scales according to your level of satisfaction.**

**How satisfied are you with the preparation you received PRIOR to your surgery (documentation / information received and meeting(s) with health professionals)...**

on the LEFT side?

Very dissatisfied Neutral Very satisfied



on the RIGHT side?

Very dissatisfied Neutral Very satisfied



If your satisfaction of the preparation you received PRIOR to the surgeries (documentation / information received and meeting(s) with health professionals) DIFFERS. Please EXPLAIN why.

____________________________________________________________________________________________________________________________________________________________________________________________________________

______________________________________________________________________________________________________

**How satisfied are you with your time at the hospital (including the anesthesia, the surgery and physiotherapy)...**

on the LEFT side?

Very dissatisfied Neutral Very satisfied



on the RIGHT side?

Very dissatisfied Neutral Very satisfied



If your satisfaction of your time at the hospital (including the anesthesia, the surgery and physiotherapy) DIFFERS between surgeries. Please EXPLAIN why.

____________________________________________________________________________________________________________________________________________________________________________________________________________

______________________________________________________________________________________________________

**How satisfied are you with the home care services your received AFTER being discharged from the hospital...**

on the LEFT side?

Very dissatisfied Neutral Very satisfied



on the RIGHT side?

Very dissatisfied Neutral Very satisfied



If your satisfaction of the home care services you received AFTER being discharge from the hospital DIFFERS between surgeries. Please EXPLAIN why.

_____________________________________________________________________________________________________________________________________________________________________________________________________________________________________________________________________________________ __________________________

**How satisfied are you with the pain management AFTER your surgery...**

on the LEFT side?

Very dissatisfied Neutral Very satisfied



on the RIGHT side?

Very dissatisfied Neutral Very satisfied



If your satisfaction of pain management AFTER your surgeries DIFFERS. Please EXPLAIN why.

____________________________________________________________________________________________________________________________________________________________________________________________________________

______________________________________________________________________________________________________

**How satisfied are you with your recovery (speed and ease of resuming your usual activities) AFTER your surgery...**

on the LEFT side?

Very dissatisfied Neutral Very satisfied



on the RIGHT side?

Very dissatisfied Neutral Very satisfied



If your satisfaction of your recovery (speed and ease of resuming your usual activities) AFTER your surgeries DIFFERS. Please EXPLAIN why.

____________________________________________________________________________________________________________________________________________________________________________________________________________

______________________________________________________________________________________________________

**How satisfied are you with your surgical wound closing method (sutures/glue/staples and bandage) ...**

on the LEFT side?

Very dissatisfied Neutral Very satisfied



on the RIGHT side?

Very dissatisfied Neutral Very satisfied



If your satisfaction on your surgical wound closing method (sutures/glue/staples and bandage) DIFFERS between surgeries. Please EXPLAIN why.

____________________________________________________________________________________________________________________________________________________________________________________________________________

______________________________________________________________________________________________________

**In general, how satisfied are you of your surgical experience as a patient...**

on the LEFT side?

Very dissatisfied Neutral Very satisfied



on the RIGHT side?

Very dissatisfied Neutral Very satisfied



If your satisfaction of your surgical experience as a patient DIFFERS between surgeries. Please EXPLAIN why.

____________________________________________________________________________________________________________________________________________________________________________________________________________

______________________________________________________________________________________________________

**How satisfied are you with the the current overall result of your joint replacement...**

on the LEFT side?

Very dissatisfied Neutral Very satisfied



on the RIGHT side?

Very dissatisfied Neutral Very satisfied



If your satisfaction of the the current overall result of your joint replacement DIFFERS between surgeries. Please EXPLAIN why.

____________________________________________________________________________________________________________________________________________________________________________________________________________

______________________________________________________________________________________________________

**For the following questions, please answer as accurately as possible.**

**For each question, you can write "Never" or "Does not apply" if this is the case for you.**

**How many DAY(S) after your surgery did you take your first shower...**

For the LEFT side?

For the RIGHT side?

**How many DAY(S) after your surgery did you start to walk without technical aid (walker/cane/crunches)...**

For the LEFT side?

For the RIGHT side?

**How many DAY(S) after your surgery did you start to climb up and down the stairs without technical aid (walker/cane/crunches)...**

For the LEFT side?

For the RIGHT side?

**How many DAY(S) after your surgery did you start to do activities of daily living (dressing, toileting, walking indoor) alone...**

For the LEFT side?

For the RIGHT side?

**How many DAY(S) after your surgery did you start to do instrumental activities of daily living (cooking, cleaning, shopping) alone...**

For the LEFT side?

For the RIGHT side?

**How many ***WEEK(S)*** after your surgery did you start to do light physical activities (cycling, swimming, walking, etc.)...**

For the LEFT side?

For the RIGHT side?

**How many ***WEEK(S)*** after your surgery did you start to do intense physical activities (running, playing tennis, skiing, etc.)...**

For the LEFT side?

For the RIGHT side?

**How many ***WEEK(S)*** after your surgery did you return to light work...**

For the LEFT side?

For the RIGHT side?

**How many ***WEEKS*** after your surgery did you return to full work...**

For the LEFT side?

For the RIGHT side?

**Recommendation**

What surgical protocol (way of doing things) between the one you had on the left side and the one you had on the right would you recommend for a hip/knee replacement?

Left


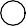

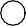

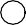


Right

No preference

Please explain why?

_____________________________________________________________________________________________________

_____________________________________________________________________________________________________

_____________________________________________________________________________________________________

_____________________________________________________________________________________________________

_____________________________________________________________________________________________________

**Suggestions**

How could we improve patients' experience for hip/knee replacement?

Please give your suggestions

_____________________________________________________________________________________________________

_____________________________________________________________________________________________________

_____________________________________________________________________________________________________

_____________________________________________________________________________________________________

_____________________________________________________________________________________________________

_____________________________________________________________________________________________________
